# Foam Stability Mediated by Cellulose Nanocrystal–Anionic Surfactant Interactions

**DOI:** 10.1021/acs.langmuir.5c06009

**Published:** 2026-02-19

**Authors:** Priscila da C. Rodrigues, Thomas Myrdek, Guilherme A. Ferreira

**Affiliations:** † Department of Physical Chemistry, Institute of Chemistry, Federal University of Bahia, Salvador, Bahia 40170-115, Brazil; ‡ Kao Chemicals GmbH, Emmerich am Rhein 46446, Germany

## Abstract

The combination of cellulose nanocrystals (CNC) and anionic alkyl poly­(ether) carboxylate surfactants offers a sustainable approach to modulating the interfacial and foaming properties of aqueous systems. This study investigated CNC–surfactant interactions in bulk and at the air–water interface using surface tension, interfacial rheology, foaming tests, particle size, zeta potential, and electron microscopy. CNC slightly increased the critical micelle concentration and reduced surfactant adsorption at the interface, indicating an interaction between the two components. Increasing the surfactant concentration improved CNC dispersion, forming smaller, more uniform aggregates. Although CNC addition slightly reduced foamability due to slower surfactant diffusion and increased surface tension, it significantly enhanced foam stability at low surfactant concentrations. This improvement was attributed to aggregated CNC forming percolated networks and reinforcing interfacial films. Dynamic surface tension and dilatational rheology confirmed an increased interfacial viscoelasticity, evidencing stronger interfacial layers. At high surfactant concentration, CNC was well dispersed, resulting in lower viscoelasticity and reduced foam stability, highlighting the importance of the CNC aggregation state. Overall, CNC–surfactant interactions enable tunable control over interfacial structure and foam stability, offering valuable insights for greener formulations in personal care, food, and household applications.

## Graphical abstract

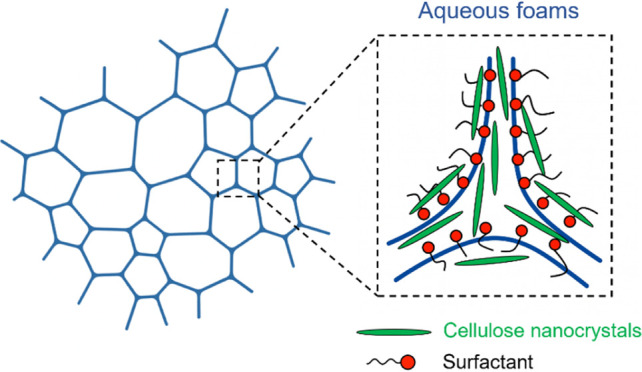

## Introduction

Cellulose nanocrystals (CNC) are rod-like nanoparticles (Figure 1a) derived from cellulose, one of the most abundant biopolymers. They typically exhibit lengths in the range of 100–500 nm and widths of 5–20 nm, with aspect ratios that allow them to impart unique properties when dispersed in aqueous medium. CNC are commonly obtained through controlled acid hydrolysis of cellulosic biomass, in which the amorphous domains are preferentially hydrolyzed, resulting in highly ordered crystalline segments with a negative surface charge.^1–3^

**Figure 1:**
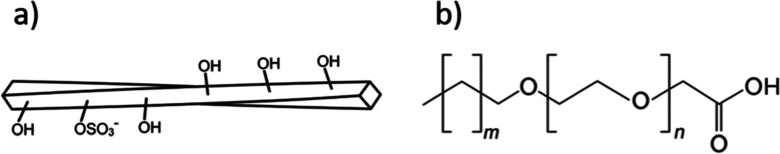
(a) Schematic representation of a CNC containing sulfate groups. (b) Chemical structure of an alkyl poly­(ether) carboxylate surfactant, in which *m* and *n* represent the alkyl chain length and the degree of ethylene oxide polymerization, respectively.

In recent years, the interfacial properties of CNC, especially at the oil–water interface, have been widely investigated, particularly for their ability to stabilize emulsions.^4–10^ Acting as solid particles that attach irreversibly at liquid–liquid interfaces, CNC can form robust interfacial layers that prevent droplet coalescence through steric and electrostatic stabilization.^11–13^ This makes them excellent candidates for Pickering stabilization, offering a sustainable alternative to conventional surfactants derived from petrochemical sources, which often raise environmental concerns due to their persistence, toxicity, or limited biodegradability. Thus, with the use of CNC, it becomes possible to design greener formulations for food, cosmetic, pharmaceutical, and environmental applications, combining functionality with sustainability.

However, due to their inherent high surface hydrophilicity, CNC exhibit intrinsically poor activity at the air–water interface,^14,15^ which hinders their capacity to stabilize aqueous foams. Consequently, their direct application in foam stabilization is generally inefficient. To address this limitation, previous studies have focused on integrating CNC with surfactants, thereby exploiting the complementary advantages of both components. For example, Czakaj and co-workers^16,17^ have investigated the interaction between sulfated and carboxylated CNC and the cationic surfactant lauroyl ethyl arginate and found that, in the presence of the surfactant, the cellulose nanocrystals became partially hydrophobized and attached at the interface, thus improving foam formation and stability at specific conditions. Similarly, other studies reported improved stability for aqueous foams when combining anionic CNC with cationic surfactants, such as hexadecyltrimethylammonium bromide (CTAB).^15,18,19^

Nevertheless, cationic surfactants are often associated with higher toxicity, potential skin and eye irritation, and limited biodegradability, which restricts their acceptance in formulations intended for consumer or environmentally sensitive applications.^20^ For this reason, increasing attention has been directed toward understanding the interactions between CNC and anionic surfactants, which are widely used in industrial and household products due to their relatively lower toxicity, cost-effectiveness, and broad availability from renewable sources.^21^

In this sense, alkyl poly­(ether) carboxylate surfactants (Figure 1b) have attracted considerable interest because they combine the characteristics of nonionic ethylene oxide (EO) blocks with the pH-responsive behavior of a weakly ionizable carboxylic acid group.^22,23^ This dual nature imparts high solubility over a wide pH range, remarkable tolerance to hard water and electrolytes, and the ability to form diverse self-assembled structures depending on the conditions. Also, they can be synthesized from starting materials derived from renewable sources, highlighting their biodegradability and low toxicity compared to conventional sulfated surfactants, which have made them important components in formulations.^24,25^ Although previous reports have demonstrated the effective interaction between CNC and traditional sulfated anionic surfactants,^19^ little is known about their interaction with other classes of anionic, biobased surfactants.

Based on these considerations, the present work proposes, for the first time, a systematic investigation of CNC–alkyl poly­(ether) carboxylate surfactant aqueous systems, focusing on their interactions both in the bulk phase and at the air–water interface. Particular attention is devoted to evaluating the foaming behavior of these mixtures, since combining the structural reinforcement provided by CNC with the interfacial activity and environmental compatibility of alkyl poly­(ether) carboxylates may open new possibilities for designing sustainable, biobased formulations with improved stability and functionality.

## Experimental Section

### Materials

The CNC, obtained by sulfuric acid hydrolysis of eucalyptus pulp, was supplied by CelluForce (Canada) in the form of a white, pulverized material. According to the supplier, the nanocrystals have an average width of 7.5 nm, an average length of 150 nm, and a crystallinity of 88%. Previous studies have reported that CelluForce CNC has an average surface charge density of 0.25 mmol of OSO_3_^–^ groups per g of material, which corresponds to approximately one sulfate group per 2–3 glucose units.^26^ The surfactant sodium lauryl poly­(ether) carboxylate, commercially known as Akypo FOAM RL 40, was kindly provided by KAO Chemicals GmbH (Germany) in the form of an aqueous solution with a solid content above 60%. According to the company, the surfactant contains on average four ethylene oxide groups, while the hydrophobic chain contains about 12 carbon atoms. Double distilled water was used in all of the experiments.

### Preparation of Aqueous CNC-Surfactant Mixtures

The stock suspension of CNC in water at a concentration of 0.6 wt % was prepared by gradually mixing the powdered material into 250 mL of water, under magnetic stirring at 500 rpm until a macroscopically homogeneous suspension was obtained. After that, stirring was continued for 24 h to ensure complete homogenization. Subsequently, the suspension was divided into five 50 mL portions, and each portion was sonicated using a probe sonicator (Ecosonic QR850) with two sonication steps of 30 s each, at 60% amplitude under a continuous pulse. A 1 min interval was allowed between each sonication step to avoid excessive heating and possible thermal degradation. Finally, all sonicated portions were combined into a single container and stored at room temperature (25 °C) for further use.

The stock surfactant solution at 10 mM was prepared by weighing the appropriate amounts of the solid and dissolving it in water under magnetic stirring for 5 min. Serial dilutions were employed to obtain other surfactant concentrations. After that, the CNC-surfactant aqueous mixtures were obtained by simply mixing equal volumes of the stock CNC suspension and the surfactant solution at the desired concentrations with the aid of magnetic stirring. In all mixtures, the CNC concentration was fixed at 0.3 wt %, while the surfactant concentration was 0.05, 0.5, or 5 mM. The final pH of all mixtures varied between 6.8 and 7.0 and was not further modified. The CNC concentration was selected based on previous reports^16,17^ in which a positive effect on foam formation and stability in aqueous surfactant solutions was observed at 0.3 wt % CNC.

### Preparation of Aqueous Foams

The foams were prepared by stirring the aqueous solutions containing the surfactant at different concentrations in the absence and presence of CNC. Volumes of 25 mL of each solution or suspension were placed in a 250 mL beaker and stirred for 30 s using a culinary mixer (XDX Mini Mixer) at a rotation speed of 10,000 rpm. Foamability was expressed in terms of the “expansion factor (*EF*)”, which relates the height of the foam formed immediately after the stirring (*h*_*0*_^*E*^) to the initial height of the liquid (*h* ^*L*^), according to

1 $$EF=\frac{h_{0}^{E}}{h^{L}}\times 100$$

The stability of the foams was evaluated by measuring the foam height at different time intervals for 4 h. From these data, plots of foam height (*h* ^E^) versus time were constructed, and linear fits of the experimental points provided the foam collapse constant rate (*k* ^E^) expressed in cm min^–1^. The higher the *k* ^E^ value, the faster the foam collapses, indicating lower kinetic stability.^27^ All measurements were performed in triplicate at room temperature, and results are presented as mean ± standard deviation.

### Characterization Methods

#### Viscosity Measurements

The apparent viscosity of surfactant solutions in the absence and presence of CNC was determined at 25 °C by using a digital viscometer (Anton Paar, model Stabinger SVM 3000). All analyses were performed in triplicate, and results are presented as the mean ± standard deviation.

### Particle Size and Zeta Potential

Nanoparticle size, expressed as the intensity-weighted mean hydrodynamic diameter (Z-avg.), was determined by dynamic light scattering (DLS), and zeta potential measurements were performed using a ZetaSizer Nano (Malvern), at room temperature, with samples placed in polystyrene cuvettes at a scattering angle of 173° and employing a 632.8 nm laser. The samples were previously diluted with distilled water in order to maximize their transparency. Both measurements were conducted in triplicate. For the zeta potential, the pH of the CNC aqueous suspensions was adjusted by adding dilute hydrochloric acid or sodium hydroxide solutions.

### Transmission Electron Microscopy (TEM)

TEM measurements were carried out for selected samples using a JEOL JEM 2800 microscope at LAMUME-IF/UFBA. Approximately 5 μL of each sample was deposited onto copper grids coated with carbon and Formvar films, and excess liquid was removed with filter paper. After 2 min, a drop of an aqueous phosphotungstic acid solution (Neon) at 1.0 wt % was placed onto the grids, fixing the cellulose nanocrystals onto the solid substrate and preventing possible aggregation during the drying process. The grids were then kept in a desiccator for at least 24 h before observation under the microscope.

### Turbidity Measurements

The turbidity of the aqueous surfactant solutions, in the absence and presence of CNC, was determined by measuring the absorbance at 500 nm of the samples placed in quartz cuvettes using a UV–vis spectrophotometer (UV-1700, Shimadzu) at room temperature and in triplicate.

### Static Surface Tension Measurements

The critical micelle concentration (cmc) of the surfactant, in the absence and presence of CNC at 0.3 wt %, was estimated from the intersection point of the straight lines that best fit the two regimes of variation of surface tension (*γ*) with surfactant concentration. For this purpose, surface tension measurements of surfactant solutions at ten different concentrations were performed in triplicate using an automated tensiometer (Sigma 701, KSV) at 25 °C, with temperature control provided by a thermostatic bath.

From surface tension measurements, the surfactant surface concentration (*Γ*) can also be estimated according to

2 $$\Gamma =\frac{-1}{RT}{\left(\frac{d\gamma }{d{\ln C}_{0}}\right)}_{T,P}$$

in which *R* is the universal gas constant, *T* is the temperature, and *C*_0_ is the bulk molar concentration of the surfactant. From Γ, the average area occupied by each surfactant molecule at the interface (*A*_mol_) can be estimated as

3 $$A_{mol}=\frac{1}{N_{A}\Gamma }$$

with *N*_A_ being the Avogadro number. The smaller the area occupied by a molecule at the interface, the greater the packing of its hydrocarbon chains, indicating that the surfactant film exhibits higher cohesion.

### Dynamic Surface Tension and Surface Viscoelastic Modulus

Dynamic surface tension measurements were performed for the aqueous solutions of the surfactant in the absence and presence of CNC using a profile analysis tensiometer (PAT-1M, SINTERFACE), equipped with a temperature-controlled measuring cell and an automatic dosing system. The drop volume was 10 μL, and surface tension values were monitored at 25 °C for ca. 300 s. The surface tension versus time (*t*) curves were fitted to the Ward–Tordai model^28,29^ to estimate the diffusion rate constant (*k*_d_) of the surfactant to the interface according to eq 4:

4 $$\gamma \left(t\right)=\gamma_{o}-k_{d}t^{1/2}$$

Subsequently, 20 area (*A*) oscillation experiments were performed at a constant frequency of 0.1 Hz and a dilatational amplitude of 10% for 50 s. Based on this, the surface viscoelastic modulus *E* can be determined according to

5 $$E=\frac{d\gamma }{d\ln A}=A_{0}\frac{d\gamma }{dA}$$

The modulus *E* is related to the surface rigidity of the bubbles. The higher its value, the more compact and cohesive the surfactant film covering the gas bubbles, generally leading to increased foam stability. The degree of overlap between the sinusoidal curves of surface area and surface tension is referred to as phase angle φ, which indicates the balance between the elastic and viscous behaviors of the surface. Lower φ values indicate a more elastic contribution, whereas higher φ values indicate a more viscous component.^27,30,31^ No variation in pH or visual aspect of the samples was observed after the measurements.

## Results and Discussion

### Aqueous Surfactant Solutions

Surface tension measurements were conducted for surfactant solutions at varying concentrations, both in the absence and presence of 0.3 wt % CNC, to determine the surfactant cmc. As shown in Figure 2, the presence of CNC resulted in higher surface tension values compared with surfactant solutions in the absence of nanoparticles. This observation suggests that, despite their anionic nature, CNC and the surfactant interact in a manner that modifies the surfactant behavior at the air–water interface. Such interactions likely decrease the number of surfactant unimers available for interfacial film formation, thereby increasing surface tension even at identical total surfactant concentrations.

**Figure 2:**
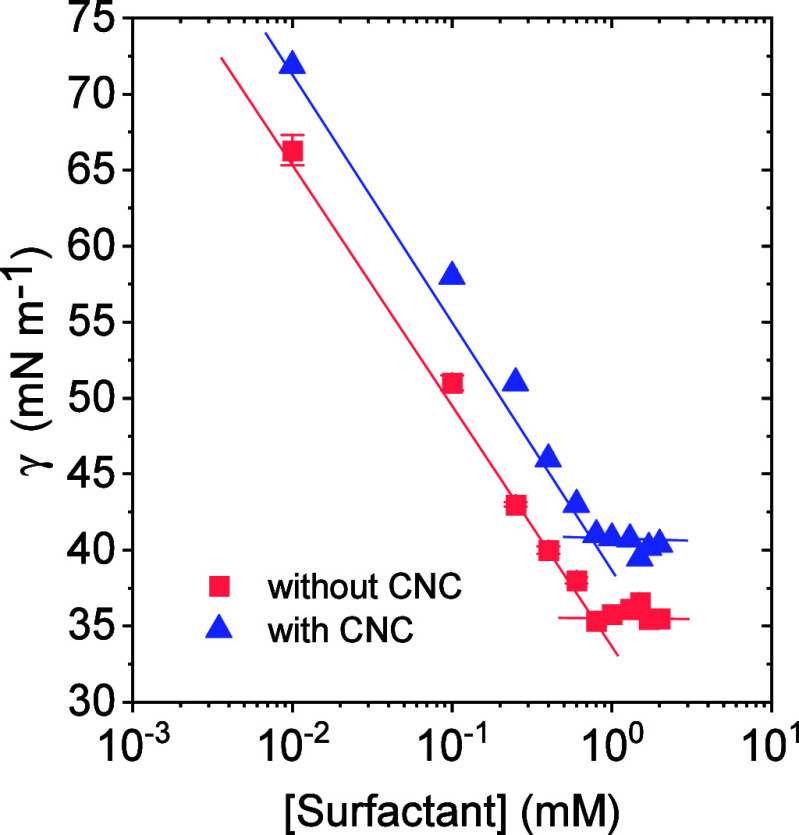
Surface tension as a function of surfactant concentration in aqueous solutions in the absence and presence of CNC at 0.3 wt %.

A similar behavior was observed by Brinatti et al.^32^ with aqueous CNC-cationic surfactant mixtures, which formed aggregates due to electrostatic interactions between the oppositely charged components. Similarly to the current work, the interaction between equal charge CNC-surfactant was suggested for sodium dodecyl sulfate (SDS), and it was attributed to the hydrophobic interactions between the surfactant alkyl chain and the nonpolar segments of the cellulose structure.^33^ Such an interaction can also take place for the surfactant under study. Also, the interaction between the ethylene oxide and carboxylate segments of the surfactant used in the current study with the hydroxyl groups of CNC through hydrogen bonds is plausible, as reported for alkyl polyglucoside surfactant mixed with CNC.^34^ However, probing such interactions in dilute aqueous systems is challenging, and therefore, they were not further investigated in the present work.

For the surfactant under study, cmc was estimated as 0.8 mM in the absence of CNC (Table 1). The obtained value is in agreement with those found in previous reports for similar surfactants. For example, Sakai et al.^35^ reported a cmc of 0.9 mM for purified C_12_O­(CH_2_CH_2_O)_4_CH_2_COONa in water, whose structure closely matches to the surfactant under study. In an opposite manner, Yue and colleagues^23^ found a cmc of 0.4 mM for an alkyl poly­(ether) carboxylate with 4 mol of EO groups but with unspecified tail length. However, issues including the purity of the sample, the pH of solution, and the heterogeneity in the alkyl chain length distribution and degree of ethylene oxide polymerization may influence these results.^22,23^

**Table 1:** Values Obtained for the Surface Properties of the Surfactant in the Absence and Presence of 0.3 wt % CNC, Namely, the Equilibrium Surface Tension (*γ_eq_*), the Critical Micelle Concentration (cmc), the Surfactant Interfacial Concentration (*Γ*), and the Area Occupied per Surfactant Molecule at the Interface (*A*_mol_)

	γ_eq_ (mN m^–1^)	cmc (mM)	Γ (mol m^–2^)	*A*_mol_ (m^2^ molecule^–1^)
without CNC	35.59 ± 1.42	0.81 ± 0.02	1.21 ± 0.02 × 10^–6^	1.38 ± 0.05 × 10^–19^
with 0.3% CNC	40.71 ± 1.10	0.90 ± 0.01	1.09 ± 0.03 × 10^–6^	1.53 ± 0.03 × 10^–19^

According to Table 1, cmc of the surfactant slightly increased (0.9 mM) upon nanocellulose addition. This shift supports the proposed surfactant–CNC interaction, as the higher cmc indicates that a greater amount of surfactant is required to reach the minimum concentration necessary for micelle formation. Surface concentration (Γ) analysis (Table 1) further corroborated this hypothesis, revealing a reduced amount of surfactant at the air–water interface in the presence of CNC, consistent with an interaction between the two components. In addition, the area occupied per surfactant molecule at the interface increased in the presence of CNC, which may indicate that CNC-surfactant aggregates may also be present at the interface to some extent or that CNC modifies the packing of the surfactant at the interface.

### Aqueous CNC Suspensions in the Absence and Presence of Surfactant

According to the TEM images of the CNC aqueous suspension in the absence of a surfactant (Figure 3), the nanocrystals were mostly agglomerated, suggesting that the dispersion process employed in the current work was not fully efficient. The nanocrystals appear to form an interconnected, entangled network rather than being uniformly dispersed, resulting in a percolated structure. The dispersion is heterogeneous, with regions of denser CNC packing interspersed with more open areas, indicating localized clustering and incomplete dispersion. Such features may influence bulk viscosity and interfacial attachment, thus playing a role in foam stabilization, and may be caused by insufficient shearing energy employed during the sonication process.^36^ Also, the absence of surfactants that usually improve the dispersibility of solid materials may have contributed to the aggregated state of the nanoparticles.

**Figure 3:**
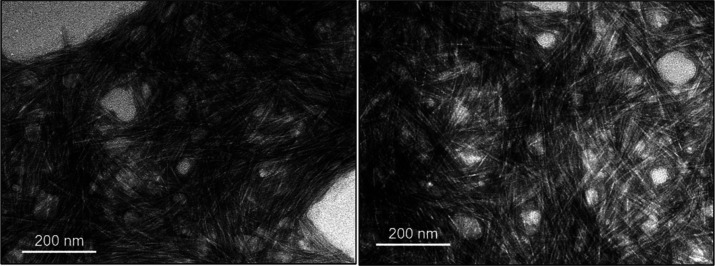
TEM images for aqueous CNC suspensions (0.3 wt %) in the absence of surfactant. Negative staining was employed. Darker areas indicate a high extent of nanocellulose aggregation.

DLS measurements of the aqueous CNC suspension revealed a broad particle size distribution with a polydispersity index (PDI) of 0.4 (Figure 4a), indicating significant size variability and corroborating the presence of aggregates not exclusively formed during sample drying for TEM. The zeta potential of CNC in the aqueous suspension was negative in the entire pH range investigated (Figure 4b), as expected from the presence of sulfate groups introduced during sulfuric acid hydrolysis of the cellulosic pulp. However, between pH 4 and 7, the zeta potential values were lower than typically recommended to ensure colloidal stability by electrostatic repulsion (commonly > |30| mV).^37^ Such reduced repulsion likely promotes CNC aggregation,^38^ highlighting the need for optimization of the dispersing process to improve the stability of both the particle suspensions and the foams studied.

**Figure 4:**
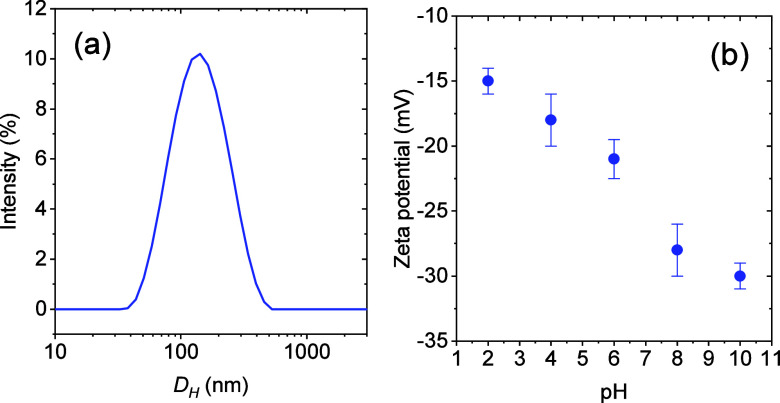
(a) Size distribution curve as obtained by DLS and (b) zeta potential as a function of pH for aqueous CNC suspensions (0.3 wt %).

In order to improve CNC dispersibility in water, the surfactant sodium lauryl poly­(ether) carboxylate was added at increasing concentrations from 0.05 to 5 mM, below and above its cmc (Table 1). The addition of surfactant in all concentrations tested promoted a decrease in the cloudiness of CNC aqueous suspensions. As shown in Figure 5a, the absorbance at 500 nm of CNC suspensions decreased with increasing surfactant concentration, which can be interpreted as evidence that surfactant addition improves the formation of smaller CNC aggregates. Considering that larger particles scatter more light and thus produce higher turbidity, the observed reduction in the absorbance values suggests that the surfactant enhances CNC dispersion by preventing the formation of large aggregates that would otherwise lead to opaque suspensions.

**Figure 5:**
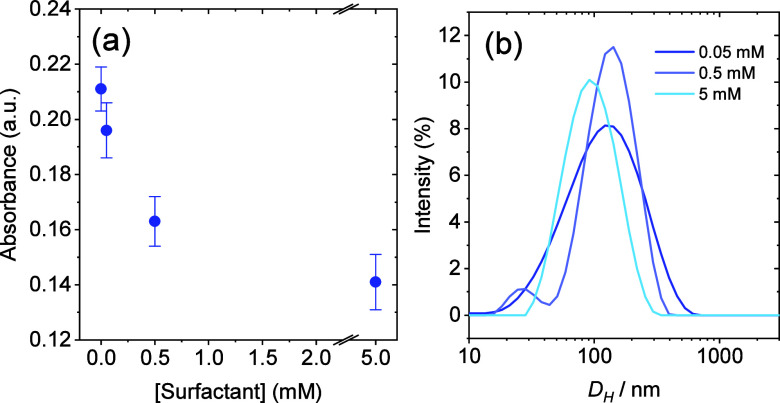
(a) Absorbance at 500 nm and (b) size distribution curves, obtained by DLS, of aqueous CNC suspensions (0.3 wt %) at different surfactant (sodium lauryl poly­(ether) carboxylate) concentrations.

The particle size distribution curves obtained by DLS (Figure 5b) corroborated the absorbance results, showing improved CNC dispersion in the presence of a surfactant. With increasing surfactant concentration, the size distributions became narrower, indicating greater particle size uniformity, with the peaks being shifted toward smaller diameters. TEM images (Figure 6) also revealed smaller aggregates, with individual nanocrystals becoming visible at the highest surfactant concentration tested.

**Figure 6:**
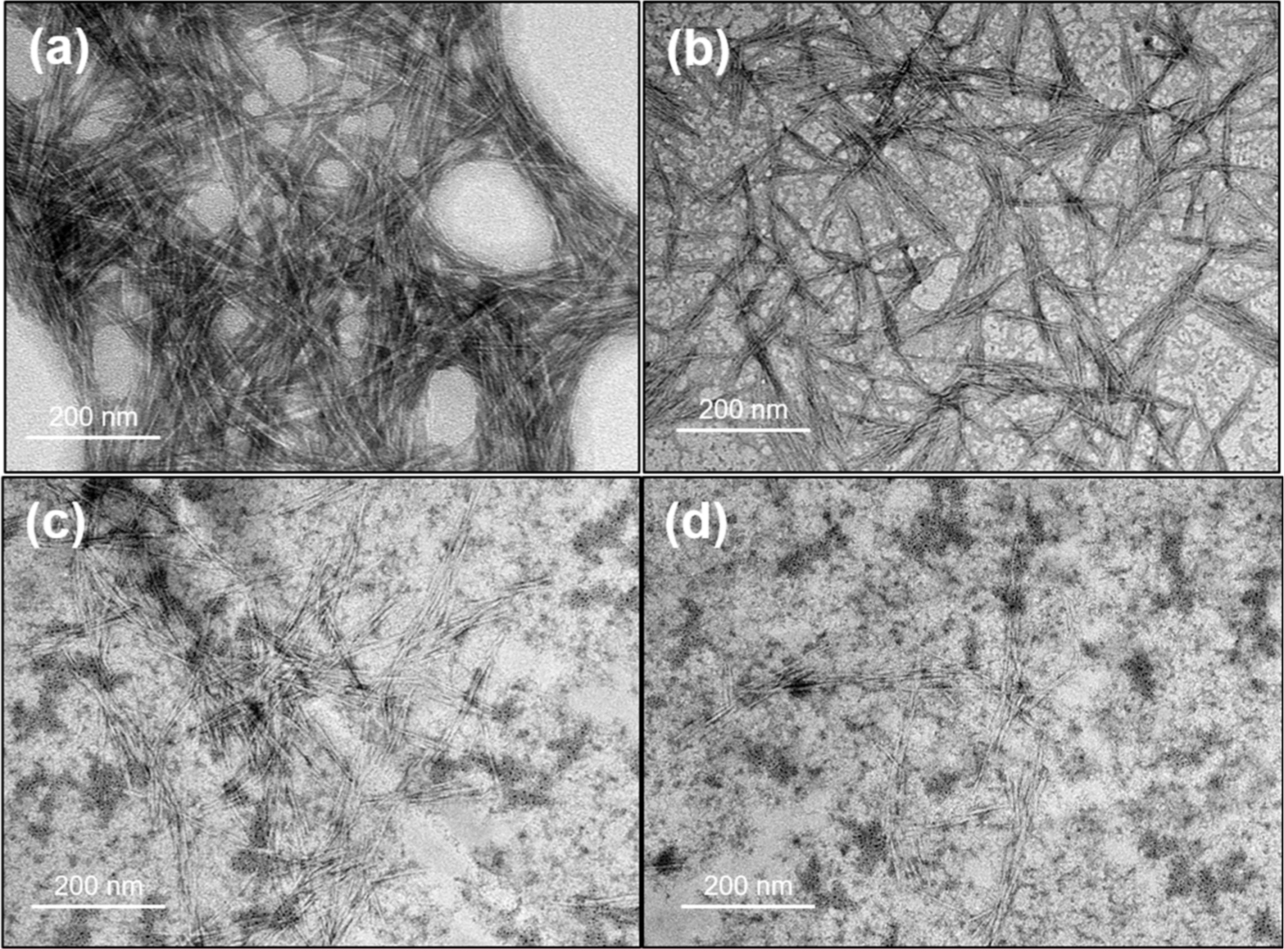
TEM images for (a) aqueous CNC suspensions (0.3 wt %) in the absence and presence of surfactant sodium lauryl poly­(ether) carboxylate at concentrations of (b) 0.05 mM, (c) 0.5 mM, and (d) 5 mM. Negative staining was employed, and the dark spots observed in panels (c,d) are attributed to the staining agent, which interacts with residual salts possibly introduced with the surfactant.

These findings suggest that surfactant addition stabilizes CNC suspensions through a progressive modification of the aggregation state. It can be considered that, initially, surfactant molecules adsorb onto cellulose particle surfaces and partially disrupt weak pre-existing CNC–CNC contacts. This adsorption increases colloidal stabilization, reducing the probability of secondary aggregation and limiting the growth of larger clusters. As a result, the system evolves toward a more homogeneous dispersion characterized by smaller, more weakly correlated CNC assemblies rather than extended aggregates.

It is important to note that all CNC suspensions were prepared using the same dispersion protocol, and the marked differences observed upon addition of the surfactant arise from CNC–surfactant interactions rather than from variations in mechanical dispersion or inherited aggregation from the starting material. It is worth noting that the distinction drawn here between mechanically dispersed and surfactant-modified CNC suspensions is based on established literature and qualitative insights rather than on a systematic, direct experimental comparison performed within this work.

For practical applications, a high degree of CNC dispersibility in a liquid medium is important since their high aspect ratio and size often promote aggregation and sedimentation, thereby limiting their performance. To overcome these challenges, several strategies have been investigated, including surface chemical modification^39^ and the use of surfactants^40^ or dispersing agents.^41^ These approaches aim to reduce interparticle interactions, enhance colloidal stability, and ensure homogeneous distribution of CNC in different solvent systems,^42^ which is essential for maximizing their interfacial properties in formulations.

As for the application of surfactants and other additives to improve dispersibility, most of the works focused on the use of oppositely charged components, such as sodium ions,^43^ quaternary ammonium salts,^40^ and arginine.^41^ However, as in the current study, previous works have also reported the improved dispersion of anionic cellulosic materials in aqueous media upon the addition of surfactants with the same charge. For example, Silva et al.^44^ observed that the addition of SDS to aqueous microfibrillated cellulose facilitated the redispersion of the freeze-dried materials, while its addition in aqueous suspensions of CNC did not cause particle aggregation.^38^

As for the apparent viscosity of the aqueous CNC-surfactant mixtures, Table S1 reveals that the presence of sodium lauryl poly­(ether) carboxylate does not improve the viscosity of CNC suspensions at the tested concentrations. This result could influence foam stability, since an increase in viscosity may decrease the rate of liquid drainage and coarsening.^45^

### Foam Formation and Stability

Regarding the foamability of the aqueous surfactant solutions, the addition of CNC led to a slight decrease in the amount of foam formed, as expressed by the expansion factor (*EF*) displayed in Figure 7. This behavior can be attributed to the higher surface tension of surfactant solutions containing CNC compared with those without the nanocrystals (Figure 2), which requires a greater energy input, or a longer agitation time, to produce the same foam volume as in surfactant solutions in the absence of CNC, which exhibited lower surface tension.

**Figure 7:**
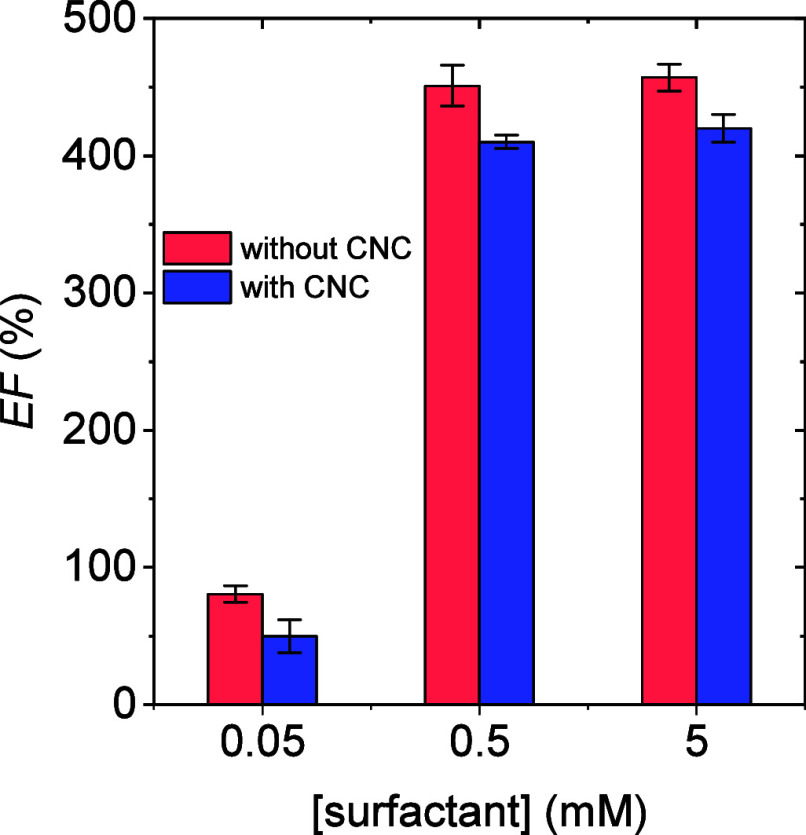
Foam expansion factor (*EF*) for aqueous surfactant (sodium lauryl poly­(ether) carboxylate) solutions at different concentrations in the absence and presence of 0.3 wt % CNC.

The viscosity of the resulting mixtures could also contribute to the obtained results since more viscous liquids require greater energy to generate the same foam volume. However, although the viscosity of the solution increased upon CNC addition, the change was negligible (Table S1), suggesting that viscosity is not the main factor responsible for the smaller expansion factors. Reduced foamability in CNC-containing systems may also result from slower surfactant diffusion to the air–water interface, necessitating longer agitation times for foam formation.

Previous reports on the foamability of aqueous cellulosic nanomaterials-surfactant mixtures have shown different results depending on the compositions tested. Xiang et al.^46^ observed that the presence of 0.3 wt % of cellulose nanofibers in aqueous SDS solutions at concentrations equal to or above the cmc slightly increased the volume of foams obtained by gas purging. Zhang et al.^47^ also reported improved foam volumes for aqueous SDS:CTAB mixtures in the presence of lignin-cellulose nanofibrils. In an opposite manner, Li and colleagues^19^ reported a decreased foam volume in aqueous CTAB solutions as the carboxylated cellulose nanofiber concentration increased.

Figure 8a–c shows the variation in foam height (*h* ^E^) as a function of time after agitation ceased. The results revealed two distinct drainage stages. The first stage was characterized by rapid liquid drainage, generally occurring within the first hour (50–60 min) after foam formation. The second stage was slower, with *h* ^E^ reaching a plateau, indicating the stabilization of the drainage process. This type of behavior has been previously reported^27^ and is associated with the existence of distinct stages in the drainage process: an initial stage, in which the liquid phase is rapidly expelled from the foam films, followed by a slower phase of residual drainage. This behavior was observed both in the absence and presence of CNC and for all surfactant concentrations tested, suggesting that CNC addition does not significantly alter the drainage stages of the foams.

**Figure 8:**
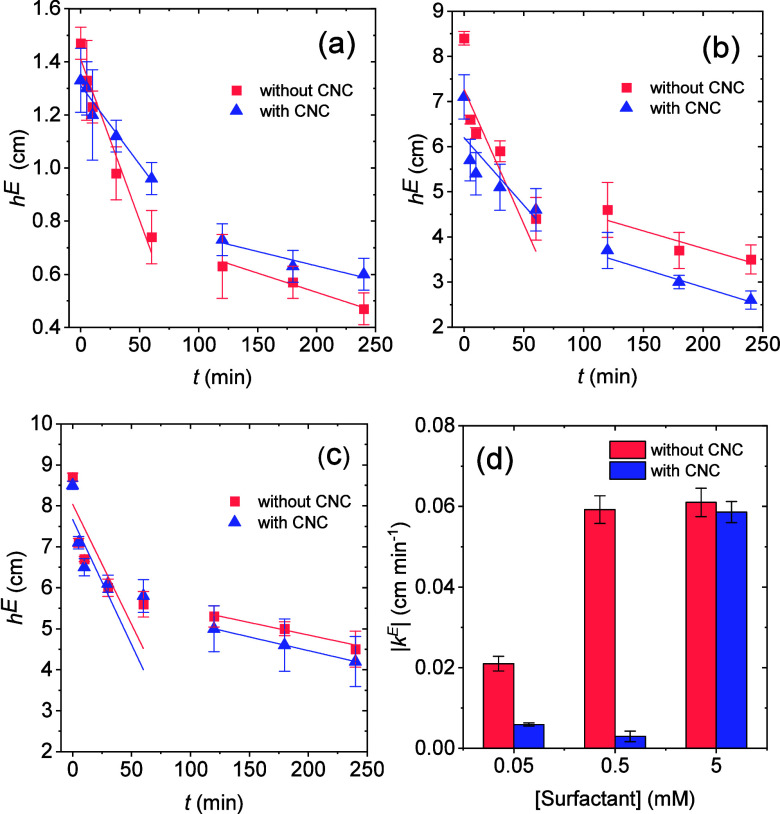
Foam height (*h* ^E^) as a function of time for different surfactant (sodium lauryl poly­(ether) carboxylate) concentrations in the absence and presence of 0.3 wt % CNC: (a) 0.05 mM surfactant, (b) 0.5 mM surfactant, and (c) 5 mM surfactant. The continuous lines are linear fits to the experimental data points at the two different foam drainage regimes. (d) Modulus of foam collapse rate constant (*k* ^E^) for the first stage of foam drainage.

To quantify this process, linear fitting was applied to each drainage regime. The slope of the fitted lines corresponds to the foam collapse rate, denoted as *k* ^E^, which represents the rate at which the foam breaks down. The *k* ^E^ values obtained for the first drainage regime are presented in Figure 8d, providing a quantitative measure of the foam stability over time. Results for the second regime are presented in Figure S1, and no significant differences in foam collapse rates were observed upon CNC addition.

For the first regime, the results indicated that the addition of CNC significantly reduced the foam collapse rate, which was substantially decreased in the first drainage stage for the two lowest surfactant concentrations (Figure 8d), leading to increased foam stability. Interestingly, at the highest surfactant concentration tested (5 mM), the presence of CNC had little effect on the foam stability. A possible explanation for these findings is that the degree of CNC aggregation positively influences the foam stability. As previously discussed, increasing the surfactant concentration improves CNC dispersion in suspension. At the two lowest surfactant concentrations, CNC remains aggregated (Figures 5 and 6), which may favor the formation of larger, network-like structures that contribute to a reduction in the foam collapse rate. In fact, previous reports have shown that particle aggregates can block the Plateau channels of a foam, thus decreasing liquid drainage.^48,49^ However, the viscosity of the surfactant solutions did not change significantly with CNC addition (Table S1), suggesting that this may not be the dominant mechanism responsible for the improvement in foam stability.

Another possible explanation is that CNC attaches to the liquid film surrounding the bubbles together with the surfactant. In this case, CNC could enhance the mechanical strength of the film, making it more rigid and less susceptible to film thinning or bubble coalescence, thereby increasing the foam stability. Hence, at low surfactant concentrations, limited surface coverage does not break CNC aggregates, thereby maintaining an interconnected structure at the bubble interface that promotes foam stability, whereas at higher surfactant concentrations, better CNC dispersion would result in the formation of a weaker interfacial film with reduced foam stability.

To gain further insight into the effect of CNC on the stability of foams formed by the surfactant solutions, dynamic surface tension measurements were performed considering it was shown to be a powerful technique to study cellulosic nanoparticles at interfaces.^50^ The results, presented in Figure 9a, confirm that in the presence of CNC, the surface tension of aqueous surfactant solutions is higher than in the absence of nanocellulose, confirming the interaction between both components. From Figure 9b, it was possible to see that the rate of change in surface tension at short time scales was slightly higher for the surfactant solution without CNC.

**Figure 9:**
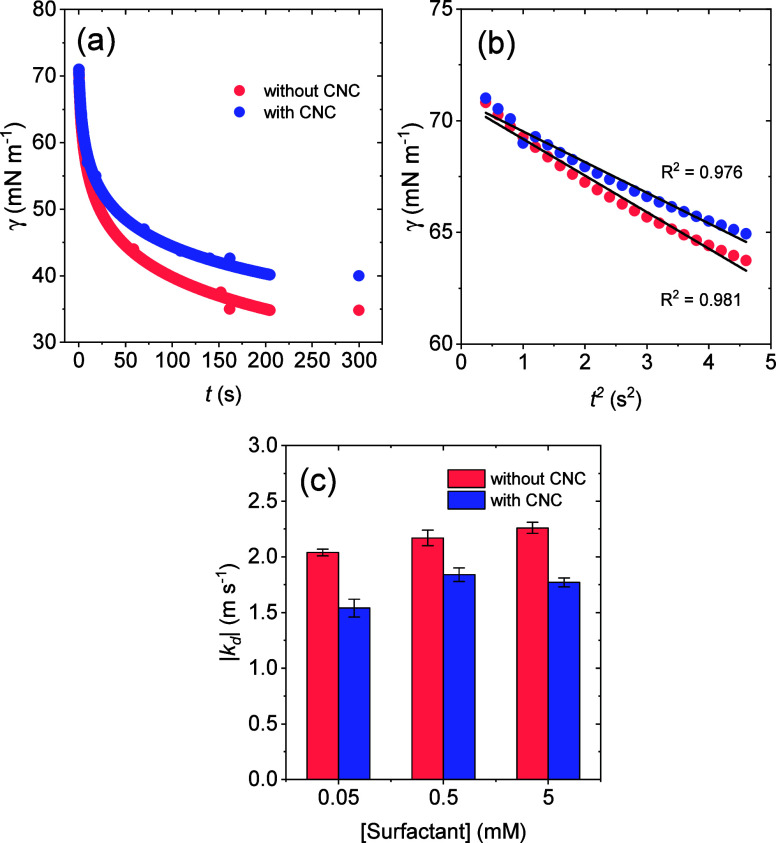
(a) Dynamic surface tension for 0.05 mM aqueous surfactant (sodium lauryl poly­(ether) carboxylate) solutions in the absence and presence of 0.3 wt % CNC. (b) Fits to experimental data at short time intervals according to eq 4. (c) Modulus of surfactant diffusion rate constants (*k*_d_) for all concentrations tested.

By fitting the experimental data points to eq 4, one can estimate the surfactant diffusion rate constants (*k*_d_), which are presented in Figure 9c and shows that upon CNC addition, *k*_d_ decreases for all surfactant concentrations tested. This finding suggests that the diffusion of surfactant molecules from the bulk solution to the surface is faster in the absence of CNC. This result is consistent with the foamability results (Figure 7), since a more rapid diffusion of surfactant molecules leads to greater foam formation under the same agitation conditions, i.e., rapid surfactant diffusion facilitates bubble coverage during agitation, resulting in the formation of a larger foam volume.^27,29^

Because previous reports have shown that the rheological properties of the liquid–gas interface are important for foam stability,^51^ the surface viscoelastic modulus (*E*) of surfactant solutions in the absence and presence of nanocellulose was investigated and the results are displayed in Figure 10a. It was seen that the *E* values for the solutions without CNC decrease slightly with an increasing surfactant concentration. This behavior is likely due to less efficient molecular packing at the interface, possibly resulting from electrostatic repulsion between negatively charged polar head groups. Such an effect suggests the formation of a less cohesive film covering the air bubbles. This observation may account for the increase in the foam collapse rate during the first drainage stage with the surfactant concentration, as shown in Figure 8d.

**Figure 10:**
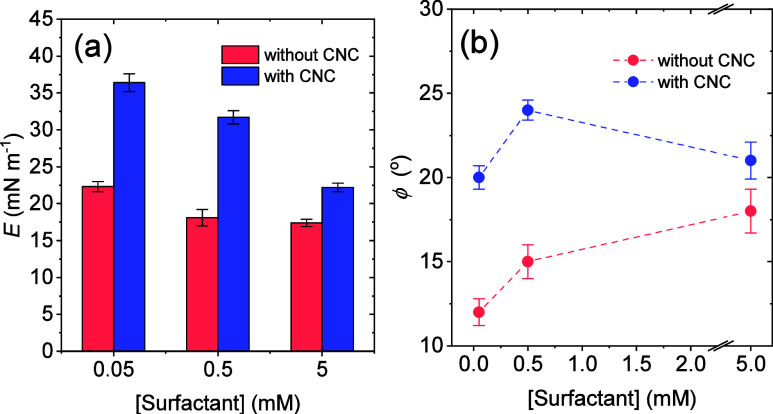
(a) Surface viscoelastic modulus (*E*) and (b) phase angle (φ) for aqueous surfactant (sodium lauryl poly­(ether) carboxylate) solutions at different concentrations in the absence and presence of 0.3 wt % CNC.

Based on this, one can suggest that as the surfactant concentration increases, the interfacial monolayer becomes less rigid, facilitating gas transfer between bubbles and accelerating foam breakage. Previous works have also reported decreased *E* values with increasing surfactant concentration, such as for sodium oleate and sodium tetradecyl sulfonate^52^ and sodium acyltaurates.^53^ The results in Figure 10a also revealed that CNC addition led to a considerable increase in the surface viscoelastic modulus, particularly at the two lowest surfactant concentrations. This finding indicates that the nanocrystals indeed participate in the formation of the interfacial film surrounding the bubbles, generating cohesive layers that enhance foam stability, in agreement with the results shown in Figure 8.

In fact, the aggregation state of CNC seems to play a key role in foam stability. At the highest surfactant concentration (5 mM), in which the nanocrystals are well dispersed, the *E* modulus was smaller (Figure 10a), which may explain the formation of foams with decreased stability under this condition. By comparing the obtained surface viscoelastic modulus values with those for other systems described in the literature, one can observe that the CNC-surfactant composite films of the current work are more stiff than those formed by conventional surfactants, such as SDS (*E* ≈ 4.0 mN m^–1^)^54^ and alkylphenol ethoxylates (between 16.3 and 24.1 mN m^–1^).^27^ Also, the interfacial films have higher *E* values than other Pickering foams, including those stabilized by starch particles,^55^ silica,^56^ and protein aggregates,^57^ which may explain the enhanced stability of the aqueous foams with CNC. Such high *E* values may be attributed to the rigidity and anisotropic shape of cellulose nanocrystals, combined with their aggregated nature, which leads to the formation of mechanically reinforced interfacial composite films. Additionally, such a high surface elastic modulus may indicate the formation of dense particle surface coverage.

The phase angle (φ) values shown in Figure 10b indicate that the addition of CNC makes the viscous component of the interfacial film more important (i.e., the angle increases when compared with surfactant solutions without nanocellulose), supporting the idea that the film becomes more rigid and viscous as the nanoparticles are added. However, at the highest surfactant concentration (5 mM), the φ value decreases, suggesting that the film becomes more elastic than at lower concentrations—further supporting the notion that well-dispersed nanocrystals contribute to reduced foam stability.

## Conclusions

This study demonstrated that the combination of cellulose nanocrystals with an anionic alkyl poly­(ether) carboxylate surfactant significantly modifies both the bulk and interfacial properties of aqueous systems. The interaction between CNC and surfactant molecules, driven by hydrophobic and hydrogen-bonding forces, leads to decreased surfactant adsorption at the air–water interface and improved CNC dispersion in the bulk phase. Foamability slightly decreased in CNC-containing systems due to higher surface tension and slower surfactant diffusion; however, foam stability was notably enhanced, particularly at low surfactant concentrations. Interfacial rheological measurements confirmed that CNC contributes to the formation of cohesive viscoelastic films, resulting in slower foam drainage and reduced coalescence. The aggregation state of CNC proved to be a determining factor: more aggregated nanocrystals strengthen interfacial films and enhance stability, whereas well-dispersed CNC at higher surfactant concentrations produced more elastic but less cohesive films.

These findings advance the understanding of CNC–surfactant interactions and provide a mechanistic basis for tuning foam performance using renewable colloidal particles. Future studies should focus on quantifying interfacial adsorption/attachment kinetics and elucidating the true nature of the aggregates by scattering techniques, enabling the rational design of sustainable foaming systems for food, cosmetic, and environmental applications.

## Supplementary Material



## Data Availability

Data will be made available on request.

## Notes

Bulk viscosity and foam collapse rate constants (PDF)
