# Supplementary material for: Foam Stability Mediated by Cellulose Nanocrystal–Anionic Surfactant Interactions
Source: Langmuir. 2026 Feb 19;42(8):6310–9. doi: 10.1021/acs.langmuir.5c06009 (PMC12961955; doi:10.1021/acs.langmuir.5c06009)
Supplement: Supplementary file 1 [file la5c06009_si_001.pdf]

Supporting information

Foam Stability Mediated by Cellulose Nanocrystal–Anionic Surfactant Interactions

Priscila da C. Rodrigues[1], Thomas Myrdek[2], and Guilherme A. Ferreira[1]*

[1]. *Department of Physical Chemistry, Institute of Chemistry, Federal University of Bahia, Salvador – BA, Brazil*

[2]. *Kao Chemicals GmbH, Emmerich am Rhein, Germany*

* ferreira.guilherme@ufba.br

Table of contents

Table S1 .... Page S2

Figure S1 .... Page S2

Table S1. Apparent viscosity (η) of aqueous surfactant (sodium lauryl poly(ether) carboxylate) solutions at different concentration in the absence and presence of CNC (0.3 wt.%). In all cases, standard deviation is below 10^{-4}.

**[Surfactant] (mM)**	**η (mPa s) without CNC**	**η (mPa s) with CNC**
0.05	0.89	0.98
0.5	0.90	0.98
5	0.93	0.99
CNC 0.3 wt.%	-	1.10

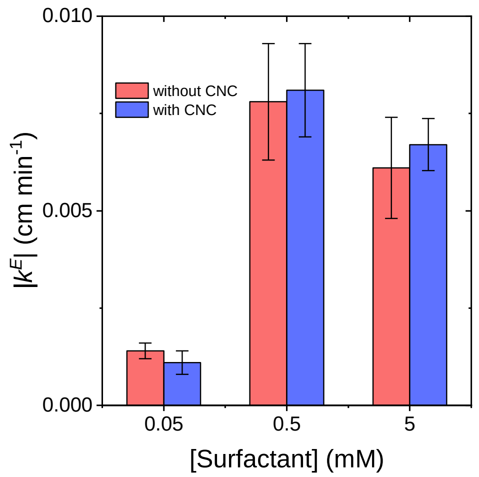

Fig. S1. Modulus of foam collapse rate constants (k^E) for foams as a function of surfactant (sodium lauryl poly(ether) carboxylate) concentration in the absence and presence of 0.3 wt.% CNC for the second stage of foam drainage.
